# Perfluoroalkyl and Polyfluoroalkyl Substances (PFAS) and Vitamin Metabolism: A Nutritional Perspective on an Emerging Environmental Health Issue

**DOI:** 10.3390/nu17101660

**Published:** 2025-05-13

**Authors:** Chen Liu, Biao Zhou, Lichun Huang, Dan Han, Mengjie He, Mengyi Zhou, Peiwei Xu, Ronghua Zhang

**Affiliations:** 1 Zhejiang Provincial Center for Disease Control and Prevention, 3399 Bin Sheng Road, Hangzhou 310051, China; 130232024301@hmc.edu.cn (C.L.); bzhou@cdc.zj.cn (B.Z.); lchhuang@cdc.zj.cn (L.H.); dhan@cdc.zj.cn (D.H.); mjhe@cdc.zj.cn (M.H.); 15058972559@163.com (M.Z.); 2 School of Public Health, Hangzhou Medical College, 481 Binwen Road, Hangzhou 310059, China

**Keywords:** perfluoroalkyl or polyfluoroalkyl substances (PFAS), vitamin metabolism, B vitamin

## Abstract

Perfluoroalkyl and polyfluoroalkyl substances (PFAS) are a class of synthetic chemicals characterized by exceptional stability and potential for bioaccumulation. Ubiquitous in the environment, PFAS can enter the human body through water, air, and dietary sources. Exposure to PFAS has been linked to various adverse health effects, including cancer, endocrine disruption, and reproductive and developmental toxicities. Emerging evidence suggests potential interactions between PFAS exposure and vitamin levels in the human body. This review provides a comprehensive understanding of the associations between PFAS and various vitamins, elucidates potential underlying mechanisms, and offers insights for the development of targeted nutritional interventions.

## 1. Introduction

Perfluoroalkyl and polyfluoroalkyl substances (PFAS) are a class of synthetic chemicals characterized by carbon–fluorine bonds, which confer exceptional stability, amphiphilicity, and thermal resistance to the PFAS structure [1]. These properties have given rise to the wide use of PFAS in food packaging, textiles, chemical industries, coatings, and paper products [2,3]. However, the high environmental persistence of PFAS [4] and their bioaccumulative nature [5] result in the retention of PFAS in ecosystems for extended periods after their release, leading to human exposure through water, food, air, and dermal contact [6]. PFAS can be classified into short-chain and long-chain compounds based on their carbon chain lengths, which may influence their bioaccumulation potential and persistence [7]. Many studies have linked PFAS exposure to a range of adverse human health effects, including cancer [8], obesity [9], reduced bone mineral density [10], immunosuppression [11], alterations in thyroid hormone levels [12], elevated cholesterol levels [13], and reproductive and developmental disorders [11,14]. These effects are believed to be mediated through several molecular mechanisms, including endocrine disruption via activation of nuclear receptors such as peroxisome proliferator-activated receptors, estrogen receptors, and thyroid hormone receptors; induction of oxidative stress and chronic inflammation; interference with lipid and glucose metabolism; epigenetic alterations; and impairment of mitochondrial function and immune signaling pathways [1]. To address growing public health concerns, regulatory agencies have established exposure guidelines for PFAS. The U.S. Environmental Protection Agency issued interim lifetime health advisory levels in 2022 of 0.004 ng/L for perfluorooctanoic acid (PFOA) and 0.02 ng/L for perfluorooctanesulfonic acid (PFOS) in drinking water [15,16], reflecting their high toxicity even at trace concentrations. Similarly, the European Food Safety Authority proposed a group tolerable weekly intake of 4.4 ng/kg body weight for the sum of four PFAS—PFOA, PFOS, perfluorononanoic acid (PFNA), and perfluorohexane sulfonate (PFHxS)—in 2020 [17]. These reference values underscore the urgent need to better understand PFAS-associated health risks and potential mitigation strategies. PFAS exposure is also known to alter vitamin levels in biological systems. Vitamins serve as crucial modulators that can mitigate the toxicity of environmental pollutants, and certain vitamins may attenuate the toxic effects of PFAS [18,19,20]. Potential underlying modulatory mechanisms include the regulation of gene expression [21], disruption of metabolic pathways [22], reduction of oxidative stress [20], and competition with PFAS for binding sites in the body [19,23].

This review systematically explores the interactions between PFAS and vitamins, highlighting recent advancements in the field. By examining the influence of different vitamins on PFAS metabolism and elucidating potential mechanisms, this work provides scientific evidence for the complex health effects of PFAS exposure and offers insights into nutritional intervention strategies.

## 2. Materials and Methods

A narrative literature review informed by a structured search was conducted in October 2024 using the PubMed and Web of Science databases to identify studies investigating the association between per- and polyfluoroalkyl substances (PFAS) and vitamin levels. The following search string was used in both databases: (PFAS OR perfluoroalkyl substances OR perfluorinated compounds OR perfluorooctanoic acid (PFOA) OR perfluorooctane sulfonate (PFOS) OR perfluorohexane sulfonate (PFHxS) OR perfluorononanoic acid (PFNA)) AND (vitamin OR vitamin A OR retinol OR vitamin D OR 25-hydroxyvitamin D OR vitamin E OR tocopherol OR vitamin B3 OR niacin OR vitamin B6 OR pyridoxine OR vitamin B9 OR folate OR folic acid OR vitamin B12 OR cobalamin OR vitamin C OR ascorbic acid). All retrieved records were imported into Zotero for reference management, and duplicates were removed. One researcher performed the initial screening of titles and abstracts, followed by a full-text review of potentially relevant articles. We excluded non-English publications, abstracts without full-text articles, conference proceedings, dissertations or theses, review articles, editorials, letters, and commentaries. A total of 22 potentially relevant studies were identified through this search and were used to inform different sections of this narrative review, including evidence synthesis and mechanistic discussion. To assess the certainty of evidence across included studies, we applied the Grading of Recommendations Assessment, Development, and Evaluation (GRADE) approach. This method evaluates the overall certainty based on five domains: risk of bias, inconsistency, indirectness, imprecision, and publication bias. According to the GRADE guidelines, animal and in vitro (cell) studies were assigned an initial overall certainty rating of “very low” due to the indirectness of the evidence. For detailed PFAS nomenclature, see Supplementary Table S1; for the complete GRADE evaluation results, refer to Supplementary Table S2.

## 3. Vitamin A

Vitamin A is a fat-soluble vitamin; the term collectively refers to all β-ionone derivatives, including retinol, retinal, retinoic acid (RA), and provitamin A carotenoids, such as β-carotene [24]. Vitamin A is essential for vision, immune function, skin integrity, and cellular growth [22]. Studies have shown that exposure to PFAS can disrupt the metabolism of vitamin A in the human body, thereby affecting its physiological functions.

Epidemiological studies suggest that exposure to long-chain PFAS, including PFOA, PFNA, and PFHxS, may disrupt retinol metabolism in humans. For example, a metabolome-wide association study conducted among 84 pregnant women in Beijing (2015–2016) reported that PFOA and PFHxS exposure was associated with elevated retinol levels and reduced concentrations of retinoic acid and 4-hydroxyretinoic acid (4-OH-RA) [25]. Similarly, a metabolomic analysis was conducted on 114 eight-year-old children from Cincinnati, Ohio, using liquid chromatography–mass spectrometry (LC–MS). For each sample, analyses were conducted using hydrophilic interaction liquid chromatography (HILIC) with an electrospray ionization (ESI) source operated in positive mode and reversed-phase chromatography (RPC) with ESI operated in negative mode, which is also referred to as C18-negative mode. The analysis results indicated that the vitamin A metabolic pathway was significantly enriched in association with serum PFNA levels under the C18-negative mode [26].

Animal studies have shown that, in addition to long-chain PFOS, its short-chain replacement, perfluorobutane sulfonate (PFBS), can disrupt vitamin A metabolism. A study investigating the metabolic toxicity of gestational PFBS exposure in maternal rats found significant alterations in the expression of genes involved in retinol metabolism [27]. Similarly, an experiment in mice demonstrated that PFOS exposure led to a marked reduction in hepatic retinol-binding protein 4 (Rbp4) levels, suggesting potential impairment in vitamin A transport [28].

The metabolic pathway of vitamin A begins with retinal, one of its three active forms. The active metabolite RA is converted by the CYP450 system to finally generate an NADPH-dependent hydroxylation metabolite [25,27]. RA plays a crucial role in embryonic development, immune regulation, and cellular differentiation [29]. PFAS may disrupt vitamin A metabolism by altering RA and its metabolites (e.g., 4-hydroxyretinoic acid [4-OH-RA]), potentially impacting growth, immune function, and overall physiological homeostasis [25]. Animal studies suggest that PFAS may interfere with retinol synthesis, transport, or metabolism by altering key metabolic pathways or gene expression [27,28]. These findings provide preliminary evidence for the PFAS-induced disruption of vitamin A homeostasis, highlighting the need for further research to elucidate the underlying mechanisms and biological implications.

## 4. Vitamin D

Vitamin D is a fat-soluble vitamin primarily obtained through sunlight exposure and dietary intake [30]. It plays a critical role in musculoskeletal health [31], immune function [32,33], hormone secretion [34], and cellular proliferation and differentiation [35]. PFAS exposure has been shown to disrupt vitamin D metabolism. A complex relationship has been observed between serum PFAS concentrations and vitamin D levels. The direction and magnitude of this association may vary depending on the specific PFAS compounds and the characteristics of the study population [35,36,37,38].

Epidemiological studies have found that PFAS levels are generally positively associated with vitamin D status in neonates and pregnant women, and an inverse association is observed in older adults, with no significant correlations reported in other populations [35,36,37,38]. One birth cohort study involving 992 mother–infant pairs conducted in Wuhan, China, found that neonatal serum concentrations of PFOS, PFHxS, and perfluorotridecanoic acid (PFTrDA) are positively correlated with total serum 25-hydroxyvitamin D (25(OH)D) levels [35]. In a cohort study of 442 African American pregnant women conducted in Atlanta, Georgia, total 25(OH)D levels were positively associated with PFOS, PFHxS, perfluorodecanoic acid (PFDA), and N-methyl perfluorooctane sulfonamidoacetic acid (NMeFOSAA) levels, while a negative correlation was observed with perfluoropentanoic acid (PFPeA); the exposures to PFHxS, PFOS, PFDA, and NMeFOSAA generally exhibited a monotonic dose–response relationship with total 25(OH)D levels, whereby higher PFAS concentrations were associated with a continuous increase in total 25(OH)D [36]. Cross-sectional studies based on the National Health and Nutrition Examination Survey (NHANES) dataset have revealed that among the general population aged 12–60 years, serum PFOS levels are negatively associated with total 25(OH)D, whereas PFHxS shows a positive correlation, When examining the dose–response relationship across PFOS quintiles, total serum 25(OH)D levels decreased monotonically with increasing PFOS concentrations. In contrast, individuals in the second to fifth quintiles of PFHxS exposure exhibited significantly higher total serum 25(OH)D levels compared to those in the lowest quintile, although no clear monotonic dose–response trend was observed [37]. In individuals over 60 years of age, serum concentrations of PFOA, PFOS, PFNA, and NMeFOSAA are inversely correlated with total 25(OH)D levels, with stronger associations observed in females compared to males in the same age group [38]. Notably, inconsistent results have been reported regarding the direction of association between PFOS or NMeFOSAA and vitamin D levels. While positive correlations have been observed in cohort studies—which are considered to provide higher levels of epidemiological evidence—negative associations were primarily reported in cross-sectional studies. However, due to differences in study populations, variations in sample sizes, and the presence of potential confounding factors, the direction in which the association is more reliable remains inconclusive. Further high-quality longitudinal studies are needed to clarify these relationships.

A potential mechanism underlying the positive association between PFAS exposure and vitamin D levels involves receptor competition and signal pathway interference [23,39]. Vitamin D exerts its biological functions by binding to the vitamin D receptor (VDR) in various tissues and organs, and its homeostasis is regulated by internal feedback mechanisms [40]. Recent studies suggest that PFAS can compete with vitamin D for VDR binding [23]. When PFAS bind to and inactivate the VDR [41], circulating total 25(OH)D levels may increase as a compensatory response. Additionally, PFAS have been identified as activators of peroxisome proliferator-activated receptor gamma (PPARγ), a key downstream mediator of vitamin D signaling [39]. PFAS binding to PPARγ may interfere with vitamin D function, leading to an upregulation of vitamin D synthesis. These mechanisms provide a possible explanation for how PFAS exposure influences circulating vitamin D levels.

**Figure 1:**
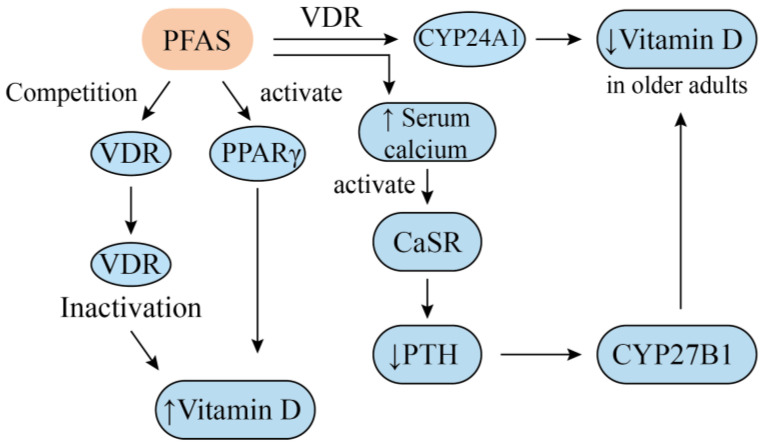
Associations between PFAS exposure and vitamin D levels. ↑—increased, ↓—decreased.

In older adults, PFAS concentrations tend to show an inverse association with vitamin D levels. This is potentially due to the upregulation of 25-hydroxyvitamin D-24-hydroxylase (CYP24A1), a key cytochrome P450 enzyme involved in hepatic 25(OH)D metabolism [42]. PFAS binding to the VDR may induce an abnormal activation of CYP24A1, accelerating the catabolism of 25(OH)D and leading to lower serum vitamin D levels. Additionally, studies have proposed an age-dependent inverse causal relationship between PFAS exposure and serum vitamin D levels, which is potentially mediated by alterations in calcium homeostasis [38]. PFAS exposure has been positively associated with serum calcium levels [43], and elevated calcium concentrations can suppress parathyroid hormone (PTH) secretion via the activation of calcium-sensing receptor (CaSR). This leads to the downregulation of CYP27B1, the key enzyme responsible for converting 25(OH)D to its active metabolite, 1,25(OH)₂D [44]. This negative feedback regulation suggests that an increase in serum calcium levels could lead to higher circulating 25(OH)D concentrations. However, in older adults, serum calcium levels are generally low and decline with age [45]. As a result, the PFAS-induced elevation in serum calcium may be less pronounced in this population, attenuating the expected feedback effect. This possibility provides a possible explanation for the age-specific association between PFAS exposure and 25(OH)D levels [38]. A schematic illustration of the proposed mechanisms is presented in Figure 1.

## 5. Vitamin E

Vitamin E is an essential fat-soluble vitamin composed of eight different tocopherol compounds, with α-tocopherol exhibiting the highest biological activity [46]. Vitamin E plays a crucial role in antioxidation, cell membrane stability, and reproductive health [47]. Metabolomic studies have indicated that PFAS exposure accelerates vitamin E metabolism in humans [48]. Additionally, animal studies suggest that vitamin E may exert a protective effect against PFAS-induced cytotoxicity, potentially through its antioxidative properties [18].

A study investigating the relationship between environmental PFAS exposure and the serum metabolome in 181 Chinese adult males found that serum PFOA was associated with increased levels of α-carboxyethyl hydroxychroman (α-CEHC) [48], a terminal metabolite of α-tocopherol (vitamin E) metabolism that has been used as an in vivo biomarker of lipid-soluble vitamin E status [49]. The elevation of α-CEHC levels suggests an accelerated breakdown of vitamin E, indicating that PFOA exposure may enhance the metabolic turnover of vitamin E in the human body.

Animal studies have also demonstrated the protective effects of vitamin E against PFAS-induced cytotoxicity. In a study investigating the impact of vitamin E on the viability of cerebellar granule neurons (CGNs) under PFAS exposure, CGNs were co-incubated with six perfluoroalkyl acids (PFAAs) and 50 mM of the lipophilic antioxidant vitamin E for 24 h. The co-treatment with vitamin E reduced the cell death induced by PFOA, PFOS, and PFNA, with the most pronounced protective effect observed against PFOA; the reduction in cell death was minimal in the PFOS and PFNA groups [18].

Because vitamin E is a lipid-soluble antioxidant, the potential mechanisms underlying the impact of PFAS on vitamin E metabolism may involve disruption of the glutathione cycle, interference with the arachidonic acid metabolic pathway, and induction of oxidative stress, which could ultimately affect vitamin E homeostasis and human health [48]. Mouse studies have also suggested that vitamin E exerts protective effects on mouse cells, reinforcing the role of oxidative stress in PFAS-induced cytotoxicity [18]. Future research should further investigate the specific effects and underlying mechanisms of the interaction between vitamin E and PFAS.

## 6. Vitamin B

### 6.1. Vitamin B3

Vitamin B3, also known as niacin or nicotinic acid, is a water-soluble vitamin that exists in vivo in two biologically active and interconvertible forms—nicotinic acid and nicotinamide [50]. It plays a critical role in energy metabolism, DNA repair, and maintenance of neurological function [51]. Metabolomic studies have indicated that PFAS exposure can disrupt vitamin B3 metabolism in humans.

A metabolomic study of 40 obese Hispanic children from urban Los Angeles identified disruptions in niacin and nicotinamide metabolism associated with plasma concentrations of PFOA, PFOS, and PFHxS, as revealed by pathway enrichment analysis [52]. These findings align with those of a separate metabolomic study that involved 114 eight-year-old children from Cincinnati, Ohio, which reported that vitamin B3 metabolism was among the enriched pathways associated with serum levels of PFOA, PFOS, PFNA, and PFHxS, as determined by hydrophilic interaction liquid chromatography (HILIC) and positive-mode electrospray ionization (ESI) mass spectrometry, together with reversed phase chromatography (RPC) and negative-mode ESI [26].

At present, however, the published studies have only established an association between PFAS exposure and vitamin B3 metabolism through pathway enrichment analysis. At the molecular level, critical gaps remain regarding how PFAS ultimately disrupt vitamin B3 regulation by interacting with key enzymes, transporters, and other molecular components involved in the vitamin B3 metabolic pathway. Future research should focus on elucidating these underlying mechanisms to provide a more comprehensive understanding of the metabolic impacts of PFAS exposure and strengthen the theoretical foundation for its biological consequences.

### 6.2. Vitamin B6

Vitamin B6 refers collectively to all 3-hydroxy-2-methylpyridine derivatives that exhibit pyridoxal bioactivity, primarily including pyridoxal, pyridoxamine, and pyridoxine [53]. As a crucial coenzyme in the synthesis of neurotransmitters, such as serotonin, dopamine, and γ-aminobutyric acid (GABA), vitamin B6 is essential for metabolic regulation and neurological health [54]. Metabolomic studies have revealed that PFAS exposure can disrupt vitamin B6 metabolism in humans.

A metabolome-wide association study of 268 African American pregnant women in Atlanta revealed that prenatal exposure to PFOS, PFNA, and PFAS mixtures affected vitamin B6 metabolism and was positively associated with maternal GABA levels, with PFNA identified as the primary driver of this effect [55].

Vitamin B6 plays a crucial role in GABA synthesis, primarily through its active coenzyme form—pyridoxal-5′-phosphate (PLP)—which facilitates the decarboxylation of glutamate via glutamate decarboxylase. As the major inhibitory neurotransmitter in the central nervous system, GABA is essential for neuronal inhibition and synaptic regulation. A potential explanation for this effect is that PFAS, particularly PFNA, may disrupt vitamin B6 metabolism, thereby influencing GABA synthesis and ultimately affecting neurological health [55]. This suggests a potential mechanistic link between PFAS exposure and neurotoxic effects. However, further experimental studies are required to validate this association and elucidate the underlying molecular mechanisms.

### 6.3. Vitamin B9

Vitamin B9, also known as folate, is an essential water-soluble vitamin that plays a crucial role in maintaining nervous system health [56] and immune function [57]. Studies have shown that folate can mitigate the adverse health effects of several environmental pollutants [58]. Current evidence suggests that PFAS exposure disrupts folate metabolism, with an inverse association observed between circulating folate levels and serum PFAS concentrations [19,59,60,61]. The potential mechanisms for this may involve glutathione (GSH) depletion and competition for cellular transporters.

Epidemiological studies have demonstrated an inverse association between folate levels and PFAS concentrations in humans, with generally consistent findings across adolescent and adult populations [19,59]. Specifically, in adolescents, red blood cell folate levels have been found to be negatively correlated with serum PFOS and PFNA concentrations, while serum folate levels show a negative association with serum PFOS concentrations. However, in adults, red blood cell folate levels have been shown to be inversely associated with serum concentrations of PFOA, PFOS, PFNA, and PFHxS, whereas serum folate levels are negatively correlated with serum PFOA, PFOS, and PFNA concentrations [19,59]. These findings suggest that folate supplementation may help mitigate the adverse effects of PFAS exposure. Furthermore, studies based on the National Health and NHANES dataset have revealed that serum PFAS exposure interferes with folate metabolic pathways, ultimately leading to an inverse association between serum PFAS concentrations and folate levels [60,61]. In adults, serum concentrations of PFOA, PFOS, PFDA, PFHxS, PFNA, and perfluoroundecanoic acid (PFUnDA) are significantly negatively correlated with red blood cell folate levels [60,61]. When PFOS exposure was categorized into tertiles, higher PFOS concentrations were associated with lower red blood cell folate and serum folate levels. Notably, perfluorocarboxylic acids with carbon chain lengths greater than eight and perfluorosulfonic acids with carbon chain lengths greater than six exhibited stronger associations with red blood cell folate levels [61]. While variations exist in specific PFAS compounds and folate biomarkers across studies, the overall trend supports a negative correlation between PFAS exposure and folate levels.

**Figure 2:**
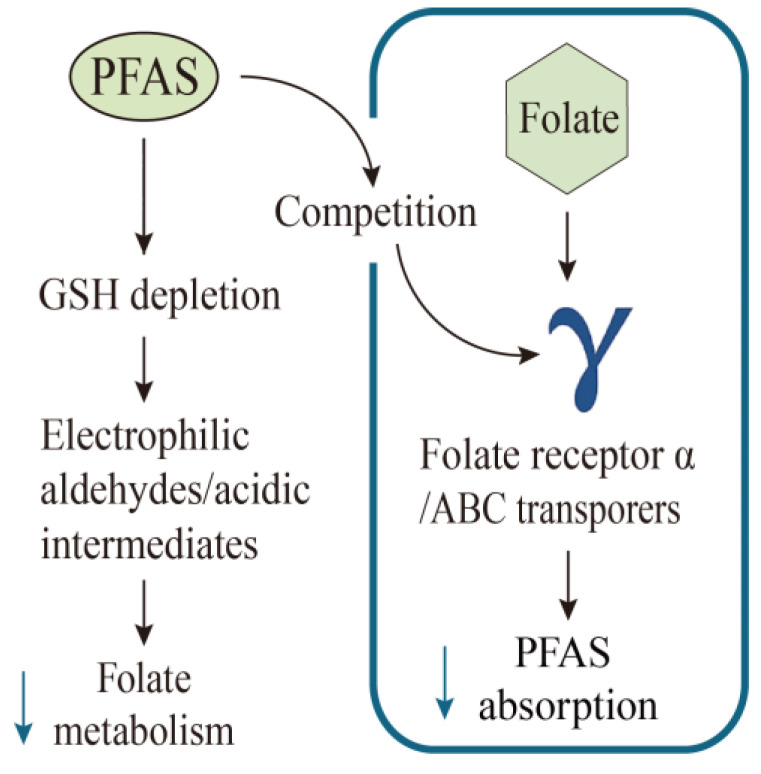
Associations between PFAS exposure and folate levels. ↓ (blue arrow)—decreased.

The biological mechanisms underlying the inverse association between PFAS exposure and serum or red blood cell folate levels remain unclear. One potential explanation is that the toxicity of bioactivated xenobiotics is often linked to GSH depletion [62]. Previous studies have suggested that PFAS toxicity may be associated with GSH depletion, which would lead to the formation of electrophilic aldehydes or acidic intermediates [63]. In particular, folate serves as a one-carbon donor in one-carbon metabolism and plays a critical role in the synthesis and metabolism of cysteine, a precursor of GSH [64], raising the possibility that PFAS may disrupt folate metabolism by depleting GSH [60]. The potential mechanism underlying the antagonistic effect of folate on PFAS could involve competition for shared transporters, including folate receptor α and ATP-binding cassette (ABC) transporters [65,66]. The competitive interaction between folate and PFAS might reduce PFAS absorption and ultimately enhance its excretion [67,68]. Figure 2 summarizes the proposed mechanistic pathways discussed above. These findings have important implications for interventions aimed at reducing the burden of PFAS in the body. Future studies should further investigate the causal relationship between folate intake and PFAS concentrations, as well as the underlying molecular mechanisms that drive this relationship.

### 6.4. Vitamin B12

Vitamin B12, also known as cobalamin, is a cobalt-containing, water-soluble vitamin that is essential for human health [69]. Vitamin B12 is a vital micronutrient with a complex molecular structure, whose unique chemical reactivity is imparted by a corrin ring coordinated to a central cobalt ion [70]. Vitamin B12 serves as a coenzyme in various enzymatic reactions and plays a critical role in DNA synthesis, red blood cell production, and neurological function [71].

Recent epidemiological research [72] has provided the first evidence of an association between PFAS exposure and vitamin B12 levels. An analysis of plasma PFAS concentrations and vitamin B12 levels in a decade-long longitudinal study following 502 adolescents and young adults (aged 12–30 years) revealed a negative correlation between vitamin B12 levels and linear PFOA, linear PFOS, and branched PFOS in both adolescents and young adults [72]. This association between vitamin B12 levels and different PFAS types suggests that the biological effects on vitamin B12 levels may be modulated by the PFAS chemical structure.

PFAS are a distinct class of halogenated compounds containing at least one fully fluorinated methyl (CF₃) or methylene (CF₂) group. Previous studies have demonstrated that vitamin B12 exhibits high catalytic activity toward various halogenated compounds [73]. For instance, vitamin B12, in combination with titanium (III) citrate [74] or other reducing agents, can effectively degrade branched PFAS [75]. Moreover, recent findings suggest that PFAS molecules preferentially interact with amino and carbonyl functional groups on the side chains of vitamin B12, stabilizing the complex through hydrogen bonding [76]. These studies indicate that influencing the bioavailability and internal burden of PFAS may potentially result in direct interactions with vitamin B12 at the molecular level.

## 7. Vitamin C

Vitamin C, also known as ascorbic acid, is a water-soluble vitamin and an essential micronutrient for humans [77]. As a potent antioxidant, it plays a crucial role in combating oxidative stress, while also supporting collagen synthesis, immune function, and neurological health [78]. Emerging evidence suggests an antagonistic interaction between vitamin C and PFAS, with vitamin C having protective effects against adverse PFAS-induced effects, such as oxidative stress, insulin resistance (IR), immunotoxicity, and leukemia-related adverse outcomes.

A double-blind, randomized crossover trial investigating the relationship between PFAS exposure, IR, and vitamin C supplementation in elderly Koreans found that PFOS and perfluorododecanoic acid (PFDoDA) levels were significantly associated with IR and oxidative stress markers. However, after vitamin C supplementation, the association between PFOS, PFDoDA, and IR disappeared [20]. These findings suggest that a diet rich in vitamin C or supplemented with vitamin C may help mitigate the adverse effects of PFAS.

Animal studies further support a protective role for vitamin C against PFAS-induced hepatotoxicity and immunotoxicity. A series of mouse model experiments, in which mice were divided into a control group, a PFAS-exposed group, and a PFAS plus vitamin C co-treatment group, demonstrated that vitamin C supplementation mitigated PFOA- and PFOS-induced liver toxicity [79,80]. Additionally, vitamin C was found to protect the spleen from PFOA-induced immunotoxicity [21].

Recent cellular studies employing network pharmacology analysis and molecular docking techniques have further revealed that vitamin C effectively inhibits PFOS-related leukemia. Key pharmacological targets identified for the protective effects of vitamin C against PFOS-associated leukemia include tumor protein p53 (TP53), mitogen-activated protein kinase 1 (MAPK1), and estrogen receptor 1 (ESR1) [81].

Current evidence remains limited regarding the antagonistic relationship between PFAS and vitamin C or the underlying mechanisms in humans. As a potent antioxidant, vitamin C exerts its protective effects by scavenging ^•^H and ^•^OH free radicals [82], and vitamin C supplementation has been shown to mitigate oxidative-stress-induced damage [83]. One proposed mechanism suggests that PFAS exposure contributes to increased insulin resistance (IR) by inducing oxidative stress, whereas vitamin C counteracts this effect by reducing oxidative stress levels, thereby alleviating PFAS-induced adverse outcomes [20]. Animal studies [21,79,80] have provided strong evidence supporting the protective effects of vitamin C against PFAS-induced toxicity. The observed hepatoprotective and immunoprotective effects of vitamin C against PFAS toxicity are apparently mediated through multiple mechanisms, including the inhibition of oxidative and endoplasmic reticulum (ER) stress, suppression of inflammatory responses [79,80], and modulation of gene expression [21]. Specifically, vitamin C mitigates PFOA-induced liver toxicity by regulating linoleic acid metabolism, inhibiting thiodiglycolic acid pathways, and promoting glutathione synthesis [79]. Its protective effects against PFOS-induced liver damage are likely mediated through enhanced hepatocyte proliferation, upregulation of FGF21 expression, and suppression of ER stress via the downregulation of inflammatory cytokines, ultimately exerting pharmacological hepatoprotective effects [80]. Furthermore, vitamin C alleviates PFOA-induced splenic immunotoxicity by modulating gene clusters, including NNT, LY6D, and LY6K, which regulate splenocyte proliferation and immune responses [21]. These findings further support the protective role of vitamin C in human health and its potential regulatory effects on environmental pollutants. However, due to physiological and metabolic differences among species, the direct extrapolation of the results from animal studies to humans remains challenging. Future research is needed to elucidate the precise mechanisms and assess the applicability of these findings in human populations.

## 8. Other Vitamins

Beyond the vitamins discussed above, research on the associations between PFAS and other micronutrients—specifically the fat-soluble vitamin K and the water-soluble B vitamins B1 (thiamine), B2 (riboflavin), and B5 (pantothenic acid)—remains limited. To date, no studies have investigated the potential relationships between PFAS and vitamin B7 (biotin).

A metabolome-wide association study involving 268 African American pregnant women in Atlanta utilized LC-MS to explore these associations. In the C18 negative ionization mode, vitamin K metabolism was associated with serum levels of PFOA, PFOS, PFNA, and PFHxS, while vitamin B1 metabolism was enriched in relation to PFOA exposure. In the HILIC mode, vitamin K metabolism showed associations with PFHxS, vitamin B2 metabolism showed associations with PFOA, and vitamin B5 metabolism showed associations with PFNA, all demonstrating pathway-level enrichment.

However, these findings stem from a single cohort study, and the directionality of the associations as well as the underlying biological mechanisms remain largely unexplored. Further epidemiological and experimental research is needed to validate these observations and clarify the potential roles of PFAS exposure in disrupting the metabolism of these lesser-studied vitamins.

## 9. Discussion

Various mechanistic pathways have been proposed to explain the associations between PFAS exposure and alterations in vitamin levels, with differences observed between fat-soluble and water-soluble vitamins.

For fat-soluble vitamins such as vitamin A and vitamin D, the most commonly discussed mechanisms involve interference with nuclear receptor signaling. PFAS may disrupt the function of receptors such as retinoic acid receptors (RARs) or the vitamin D receptor (VDR), thereby altering the transcriptional regulation of vitamin-responsive genes. In particular, competitive binding to VDR has been suggested, which may affect calcium homeostasis and immune modulation through downstream signaling interference.

In contrast, water-soluble vitamins appear to be affected through distinct metabolic or oxidative pathways. For example, studies have linked PFAS exposure to potential disruptions in one-carbon metabolism, particularly involving folate (vitamin B9) and vitamin B12, which may manifest as altered homocysteine levels or impaired methylation capacity. These disruptions can influence DNA synthesis, repair, and epigenetic regulation. Additionally, vitamin C, known for its antioxidant role, may be impacted by PFAS-induced oxidative stress. Increases in reactive oxygen species (ROS) could lead to greater consumption or degradation of vitamin C, resulting in lower circulating concentrations.

While these mechanisms are largely inferred from a limited number of experimental or observational studies, the current evidence suggests that PFAS may interfere with vitamin metabolism through both receptor-mediated and oxidative stress pathways. However, the available research remains fragmented and vitamin-specific, and few studies have attempted systematic comparisons across different vitamin classes. Moreover, certain vitamins such as vitamin E and other B vitamins have been sparsely studied, and mechanistic insights into their interactions with PFAS are lacking.

In conclusion, although the proposed mechanisms differ depending on the vitamin involved, some convergent pathways, such as nuclear receptor interference and oxidative stress, may underlie PFAS–vitamin interactions. Future mechanistic research is needed to clarify these pathways across diverse vitamins, ideally integrating molecular, cellular, and in vivo data to identify common versus vitamin-specific effects.

To our knowledge, this review provides the first comprehensive evaluation of the existing evidence regarding the interactions between PFAS and vitamins by systematically assessing the credibility and consistency of findings across epidemiological, animal, and cellular studies. A key strength of this review lies in its comprehensive summary of the associations between various vitamins and PFAS, along with its in-depth exploration of the potential underlying mechanisms. As summarized in Table 1, existing studies have reported associations between PFAS exposure and changes in vitamin levels. The table provides an overview of these findings, detailing the specific PFAS compounds investigated, the direction of associations, the study types, the references supporting each relationship, and the strength of evidence. The strength of evidence was categorized as strong, moderate, limited, or weak. This classification was made with reference to the evidence hierarchy proposed by the Centre for Evidence-Based Medicine, considering factors such as study design, sample size, and consistency across studies. Specifically, associations supported by multiple human studies with large sample sizes and consistent findings were categorized as strong; those supported by at least one large or multiple moderate-sized human studies were labeled moderate; associations from one or two small human studies or inconsistent results were considered limited; and findings based solely on animal or cell studies were classified as weak.

However, this review has certain limitations. One is that it includes only published studies, excluding gray literature or studies lacking sufficient quantitative data. Another is that the discussion on the underlying mechanisms of some vitamins remains limited due to the scarcity of relevant research. Lastly, the scope of the vitamins covered in this review is not exhaustive. For instance, current analyses of other B vitamins and vitamin K are limited and the potential associations and mechanisms remain unclear; therefore, these vitamins were not included in our review.

## 10. Conclusions

In summary, PFAS exposure shows close associations with vitamin metabolism, with potentially adverse influences on vitamin synthesis, transport, and function through mechanisms involving metabolic pathway disruption, gene expression modulation, oxidative stress, and receptor competition. While existing studies provide preliminary evidence for these PFAS effects, certain limitations remain, including the challenges in establishing causal relationships in epidemiological studies, the limited ability to extrapolate animal model findings to human health effects, and the heterogeneity across the available studies. Future research should integrate multi-omics approaches to elucidate the precise mechanisms by which PFAS affect vitamin metabolism and explore potential nutritional interventions to mitigate the health risks associated with PFAS exposure. Such efforts will contribute to a stronger scientific foundation for public health policymaking.

## Figures and Tables

**Table 1:** Associations between PFAS exposure and vitamin levels.

Vitamins	PFAS	Association Direction	Type of Study	Sample Size	Number of Studies	Reference	Strength of Evidence
Vitamin A	PFOA	↓	Metabolomics	84	1	[25]	limited
	PFOS	↓	Animal (mouse)	N/A	1	[28]	weak
	PFHxS	↓	Metabolomics	84	1	[25]	limited
	PFNA	↓	Metabolomics	114	1	[26]	limited
	PFBS	↓	Animal (rat)	33	1	[27]	weak
Vitamin D	PFOA	↓	Cross-sectional	3853	1	[38]	limited
	PFOS	Mixed (↑/↓)	Cross-sectional, Cohort	12,327	4	[35,36,37,38]	moderate
	PFHxS	↑	Cross-sectional, Cohort	8474	3	[35,36,37]	strong
	PFNA	↓	Cross-sectional	3853	1	[38]	limited
	PFDA	↑	Cohort	442	1	[36]	limited
	PFTrDA	↑	Cohort	992	1	[35]	limited
	PFPeA	↓	Cohort	442	1	[36]	limited
	NMeFOSAA	Mixed (↑/↓)	Cross-sectional, Cohort	4295	2	[36,38]	moderate
Vitamin E	PFOA	↓	Metabolomics, Animal (rat)	181 (human) N/A (animal)	2	[18,48]	moderate
	PFOS	↓	Animal (rat)	N/A	1	[18]	weak
	PFNA	↓	Animal (rat)	N/A	1	[18]	weak
Vitamin B3	PFOA	↓	Metabolomics	154	2	[26,52]	limited
	PFOS	↓	Metabolomics	154	2	[26,52]	limited
	PFHxS	↓	Metabolomics	154	2	[52]	limited
	PFNA	↓	Metabolomics	114	1	[26]	limited
Vitamin B6	PFOS	↓	Metabolomics	268	1	[55]	limited
	PFNA	↓	Metabolomics	268	1	[55]	limited
Vitamin B9	PFOA	↓	Cross-sectional	16,908	3	[19,59,61]	strong
	PFOS	↓	Cross-sectional	20776	4	[19,59,60,61]	strong
	PFHxS	↓	Cross-sectional	16,908	2	[19,61]	moderate
	PFNA	↓	Cross-sectional	20,055	3	[19,59,61]	strong
	PFDA	↓	Cross-sectional	7012	2	[60,61]	moderate
	PFUnDA	↓	Cross-sectional	6291	1	[61]	limited
Vitamin B12	PFOA	↓	Cohort	502	1	[72]	limited
	PFOS	↓	Cohort	502	1	[72]	limited
Vitamin C	PFOA	↓	Animal (mouse)	56	2	[21,79]	weak
	PFOS	↓	Animal (mouse), Cell, RCT	141 (human) 40 (animal)	3	[20,80,81]	moderate
	PFDoDA	↓	RCT	141	1	[20]	moderate

PFOA, perfluorooctanoic acid; PFOS, perfluorooctane sulfonate; PFHxS, perfluorohexane sulfonate; PFNA, perfluorononanoic acid; PFBS, perfluorobutane sulfonate; PFDA, perfluorodecanoic acid; PFTrDA, perfluorotridecanoic acid; PFPeA, perfluoropentanoic acid; NMeFOSAA, N-methyl perfluorooctane sulfonamidoacetic acid; PFUnDA, perfluoroundecanoic acid; PFDoDA, perfluorododecanoic acid. Metabolomics, metabolomics study; Cross-sectional, cross-sectional study; Cohort, cohort study; Animal (mouse/rat), animal experiment (mouse/rat); Cell, cell experiment; RCT, randomized controlled trial. ↑—increased, ↓—decreased. N/A—not available.

## Supplementary Materials

The following supporting information can be downloaded at: https://www.mdpi.com/article/10.3390/nu17101660/s1, Table S1: Classification and abbreviations of PFASs used in this review. Table S2: GRADE assessment of the certainty of evidence across included studies.
