# Supplementary material for: Perfluoroalkyl and Polyfluoroalkyl Substances (PFAS) and Vitamin Metabolism: A Nutritional Perspective on an Emerging Environmental Health Issue
Source: Nutrients. 2025 May 13;17(10):1660. doi: 10.3390/nu17101660 (PMC12113770; doi:10.3390/nu17101660)
Supplement: Supplementary file 1 [file nutrients-17-01660-s001.zip › Table S1.pdf]

Table S1. Classification and Abbreviations of PFASs Used in This Review.

Category	Abbreviation	Full Name
Short-chain Perfluoroalkyl Carboxylic Acids (PFCAs)	PFPeA	Perfluoropentanoic acid
Long-chain Perfluoroalkyl Carboxylic Acids (PFCAs)	PFOA	Perfluorooctanoic acid
	PFNA	Perfluorononanoic acid
	PFDA	Perfluorodecanoic acid
	PFUnDA	Perfluoroundecanoic acid
	PFDoDA	Perfluorododecanoic acid
	PFTrDA	Perfluorotridecanoic acid
Short-chain Perfluoroalkyl Sulfonic Acids (PFSAs)	PFBS	Perfluorobutanesulfonic acid
Long-chain Perfluoroalkyl Sulfonic Acids (PFSAs)	PFOS	Perfluorooctane sulfonic acid
	PFHxS	Perfluorohexane sulfonic acid
Sulfonamidoacetic Acids	NMeFOSAA	N-methyl perfluorooctane sulfonamidoacetic acid
